# Supplementary material for: Rational strain engineering of single-atom ruthenium on nanoporous MoS2 for highly efficient hydrogen evolution
Source: Nat Commun. 2021 Mar 16;12:1687. doi: 10.1038/s41467-021-21956-0 (PMC7966786; doi:10.1038/s41467-021-21956-0)
Supplement: Supplementary file 1 — Supplementary Information [file 41467_2021_21956_MOESM1_ESM.pdf]

Supplementary Information for

Rational strain engineering of single-atom ruthenium on nanoporous MoS_2 for highly efficient hydrogen evolution

Jiang et al.

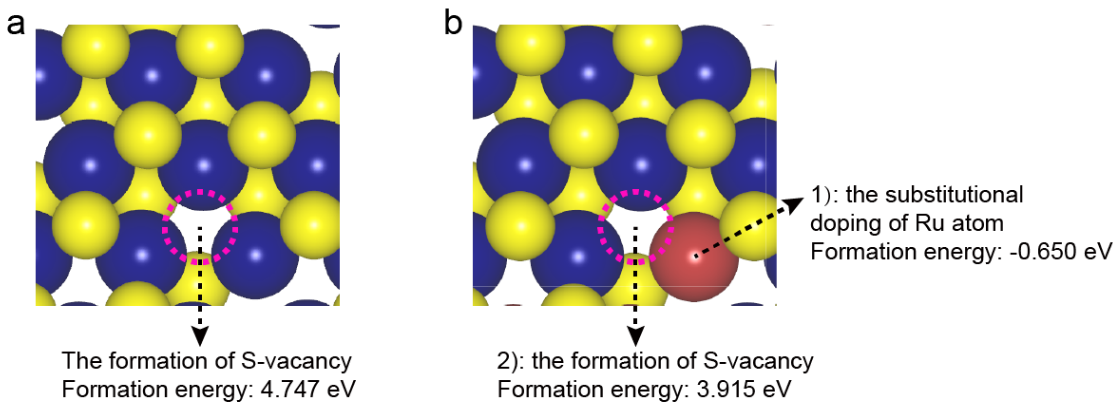

Supplementary Figure 1. The calculation of formation energy.

(**a**) Formation energy of S-vacancy in 1T-MoS_2. (**b**) 1) Formation energy of Ru atom replaces Mo site. 2) Formation energy of S-vacancy in Ru/1T-MoS_2.

The formation energy (E_f) of Ru atom replaces Mo site was calculated as following equation:

$$E_f = E_{doped} - E_{perfect} + (\mu_{Mo} - \mu_{Ru})$$

Where, E_{doped}, $E_{perfect}$, μ_{Mo}, and μ_{Ru} are the total energy for MoS_2 with Ru doped, the total energy for perfect MoS_2, the chemical potential for Mo, and the chemical potential for Ru, respectively.

The formation energy (E_f) for S-vacancy in 1T-MoS_2 was calculated as following equation:

$$E_f = E_{SV} + \mu_S - E_{perfect}$$

Where, E_{SV}, μ_S, and $E_{perfect}$ are the total energy for MoS_2 with S-vacancy, the chemical potential for S, and the total energy for perfect MoS_2, respectively.

The formation energy (E_f) for S-vacancy in Ru/1T-MoS_2 was calculated as following equation:

$$E_f = E_{Ru/SV} + \mu_S - E_{Ru/perfect}$$

Where, $E_{Ru/SV}$, μ_S, and $E_{Ru/perfect}$ are the total energy for Ru/1T-MoS_2 with S-vacancy, the chemical potential for S, and the total energy for perfect Ru/1T-MoS_2, respectively.

In the formation energy calculations, we need to calculate the chemical potential of Mo, Ru, and S atoms. In this paper, the calculated chemical potentials are equal to the DFT total energies of their ground states (**Ref. 1**).

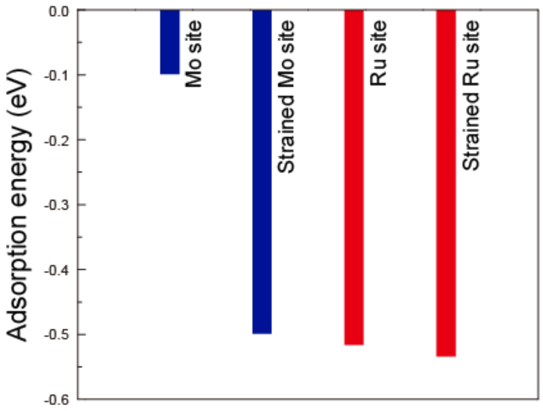

Supplementary Figure 2. The calculations of H_2O adsorption energy.

The calculations of H_2O adsorption energy at different sites on the surface of Ru/MoS_2 before and after the applied strain.

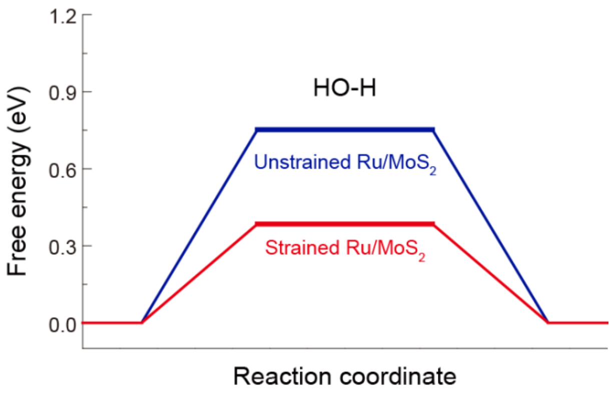

Supplementary Figure 3. DFT calculations.

Calculated free energy profiles of alkaline HER.

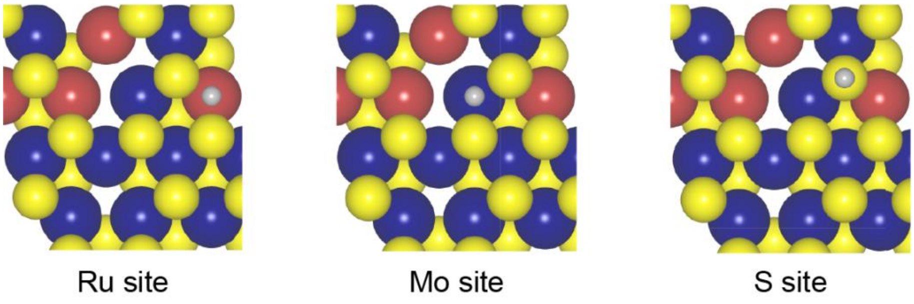

Supplementary Figure 4. DFT model.

H adsorption on different atom sites of Ru/MoS_2.

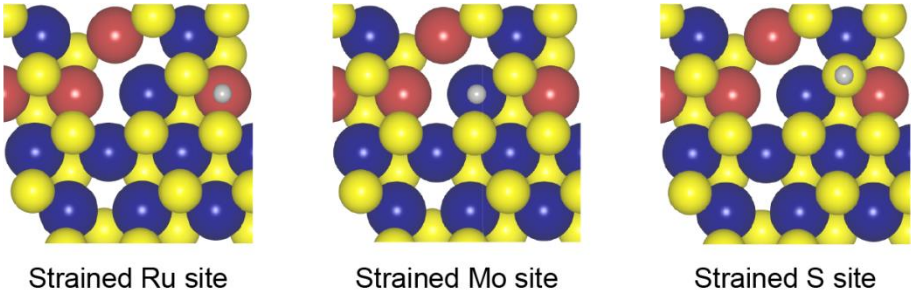

Supplementary Figure 5. DFT model.

H adsorption on different atom sites of strained Ru/MoS_2.

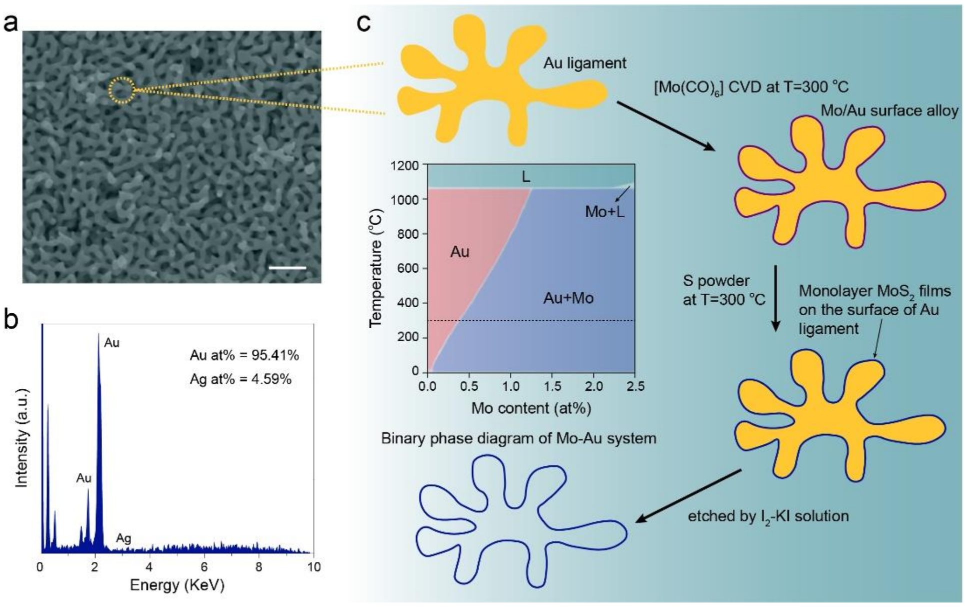

Supplementary Figure 6. Schematic illustration of the preparation of np-MoS_2.

(**a**) SEM image of nanoporous Au. (**b**) Corresponding EDS spectrum. Inset: the compositions of nanoporous Au result from EDS analyses. (**c**) Schematic illustration of the preparation of np-MoS_2. Scale bar: 200 nm.

NPG were prepared by chemically dealloying (**Supplementary Fig. 6a**) (**Ref. 2**). Specifically, the $Au_{35}Ag_{65}$ alloy were etched by HNO_3 (69 vol.%) at room temperature for 10 h, in which the Ag component were selectively leach while Au were remained to form the nanoporous structure (**Supplementary Fig. 6b**). The as-prepared NPG were washed three times with water to remove the residual acid in the nanopore channels. Afterwards, we use the NPG as a support on which $Mo(CO)_6$ vapor decomposes to form the Mo/Au surface alloy (**Supplementary Fig. 6c**) (**Ref. 3**). Under atmospheric pressure, the sulfidation of Mo/Au surface alloy were occurred by using S powder as a sulfur source. After sulfidation, a few layers of MoS_2 were group up on the surface of NPG to form the MoS_2-NPG hybrid materials. Finally, the np-MoS_2 were obtained by etching the Au component with I_2-KI solution.

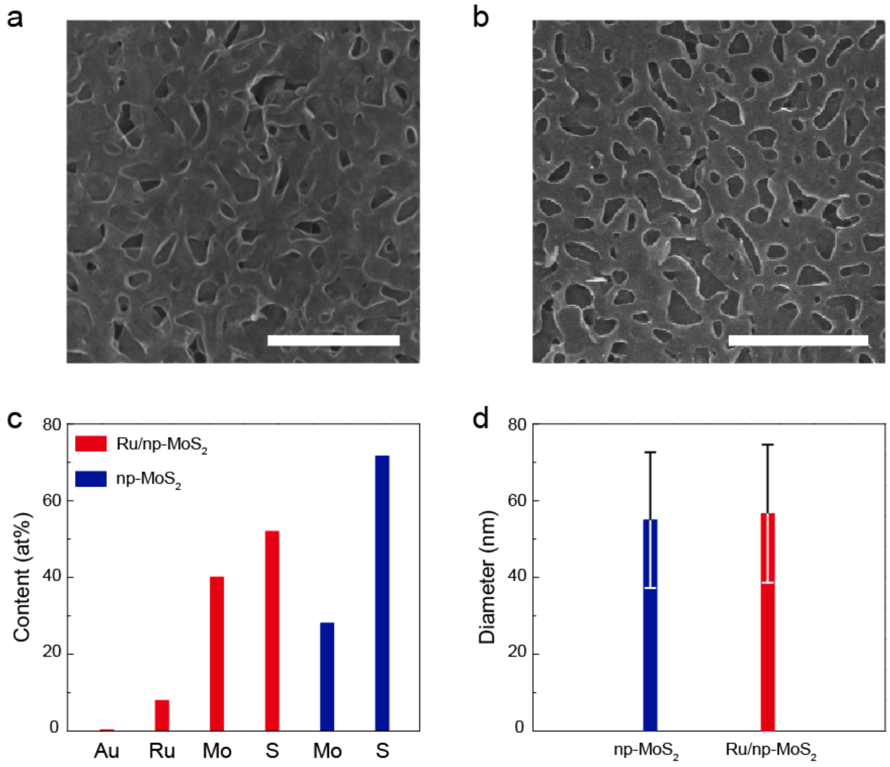

Supplementary Figure 7. SEM characterizations of np-MoS_2 and Ru/np-MoS_2.

SEM images of np-MoS_2 (**a**) and Ru/np-MoS_2 (**b**). (**c**) The compositions of np-MoS_2 and Ru/np-MoS_2 result from EDS analyses. (**d**) The average diameters of ligament for np-MoS_2 and Ru/np-MoS_2. Error bars represent the standard deviation from multiple measurements. Scale bars: (a, b) 500 nm.

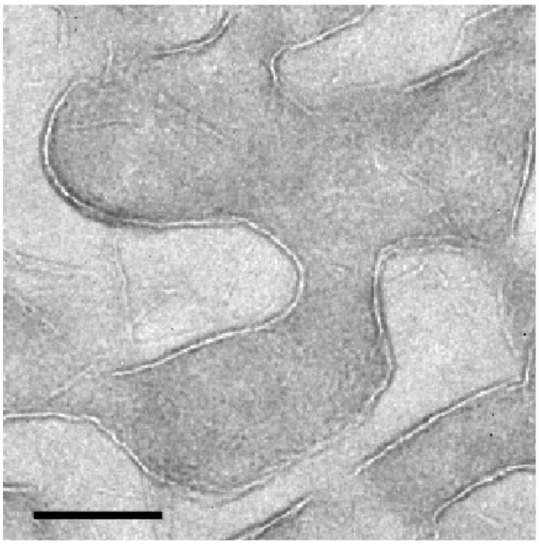

Supplementary Figure 8. TEM characterizations of Ru/np-MoS_2.

TEM image of Ru/np-MoS_2, showing the nanotube-shaped ligaments. Scale bar: 100 nm.

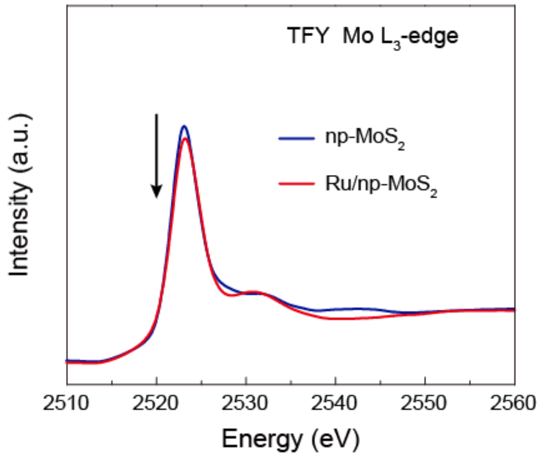

Supplementary Figure 9. XAS characterizations of np-MoS_2 and Ru/np-MoS_2.

Mo L_3-edge XANES spectra of np-MoS_2 and Ru/np-MoS_2.

The Mo L_3- and S K-edges (**Fig. 2e**) XANES spectra were measured by using the Lytle detector under TFY mode.

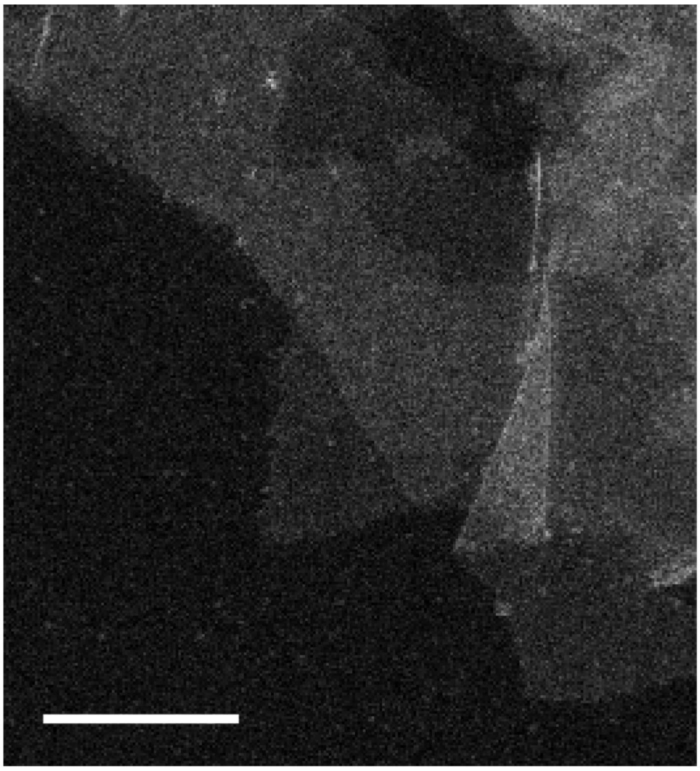

Supplementary Figure 10. HAADF-STEM characterizations of Ru/P-MoS_2.

HAADF-STEM image of Ru/P-MoS_2, showing a few layers of MoS_2. Scale bar: 50 nm.

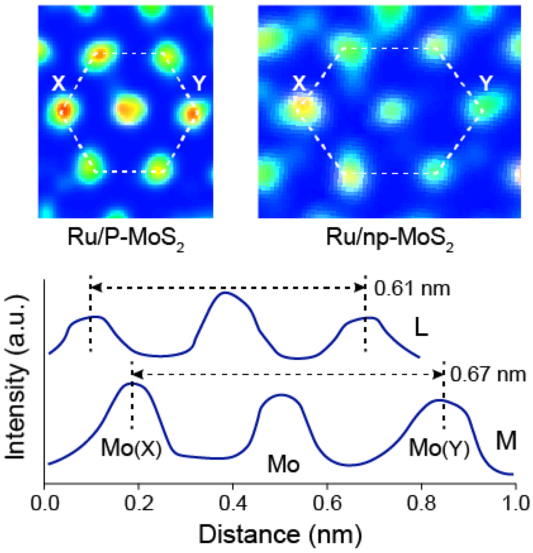

Supplementary Figure 11. HADF-STEM characterizations of strain.

Representative HAADF-STEM images at unstrained region (Ru/P-MoS_2) and strained region (Ru/np-MoS_2). Their intensity profile analyses were shown below the images.

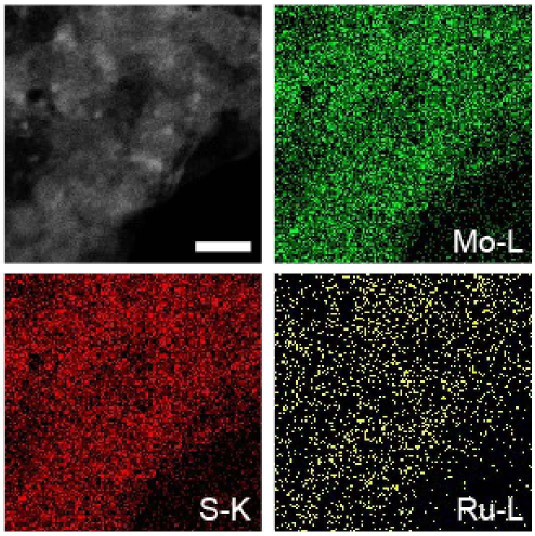

Supplementary Figure 12. STEM-EDX element mapping.

STEM image and corresponding STEM-EDX element mapping of Ru/np-MoS_2. Scale bar: 100 nm.

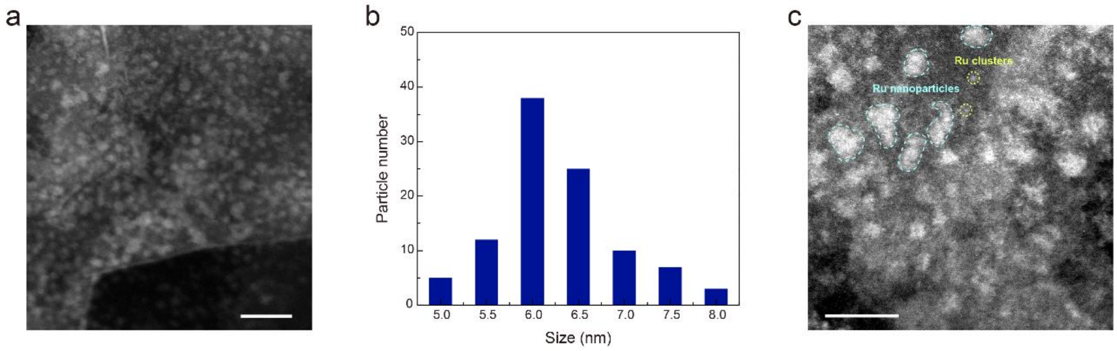

Supplementary Figure 13. HAADF-STEM characterizations of Ru_{NP}/np-MoS_2.

HAADF-STEM image of Ru_{NP}/np-MoS_2 (**a**), showing that Ru nanoparticles are uniformly distributed on the ligament of np-MoS_2 with an average diameter of ~6.25 nm (**b**). Magnified HAADF-STEM image of Ru_{NP}/np-MoS_2 (**c**), showing many Ru nanoparticles and clusters on the surface of MoS_2. Scale bars: (a) 20 nm, (b) 10 nm.

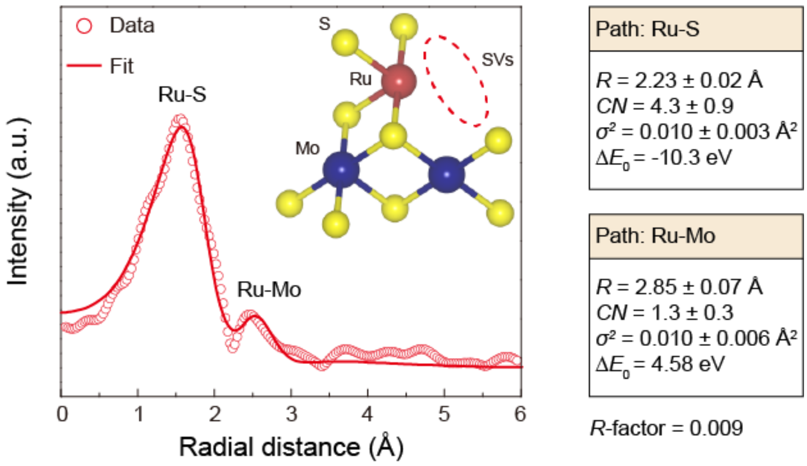

Supplementary Figure 14. FT-EXAFS fitting curves of Ru/np-MoS_2.

The local atomic structure of Ru in Ru/np-MoS_2 derived by EXAFS fitting matches well with the Ru-S4 model, suggesting the loss of two S atoms around Ru atom thus forming the SVs. The emerge of Ru-Mo bond with a bond length of 2.85 Å in Ru/np-MoS_2 indicates the substitutional doping of Ru atom in 1T-MoS_2 (**Ref. 4**).

Note: R represents the interatomic distance; CN represents the coordination number; σ^2 represents the Debye-Waller factor; ΔE_0 represents the edge-energy shift.

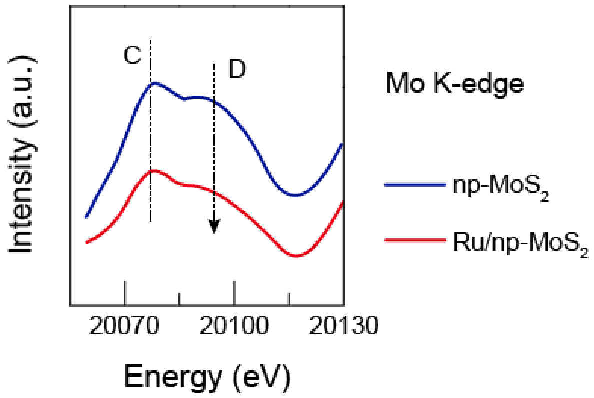

Supplementary Figure 15. Spectral features of Mo K-edge XANES spectra.

Spectral features of Mo K-edge XANES spectra of np-MoS_2 and Ru/np-MoS_2.

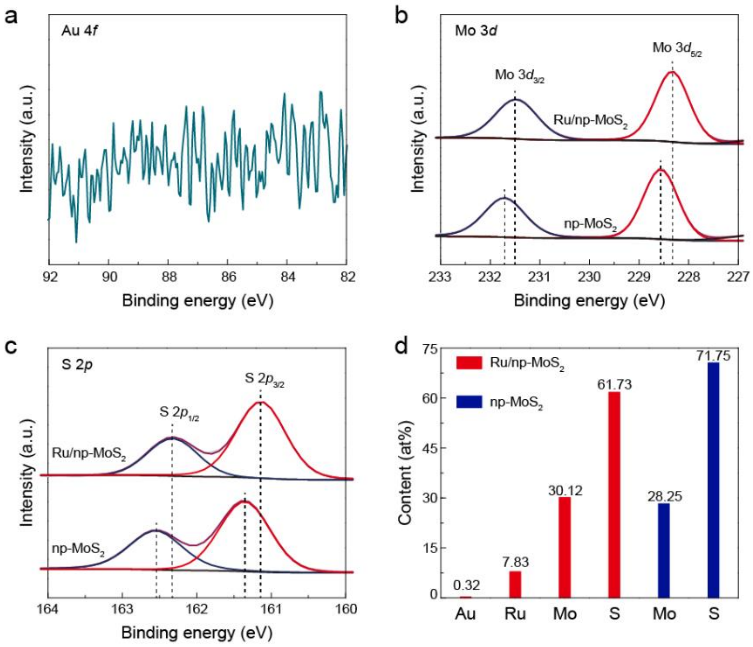

Supplementary Figure 16. XPS characterizations of np-MoS_2 and Ru/np-MoS_2.

High-resolution Au 4*f* (**a**), Mo 3*d* (**b**), and S 2*p* (**c**) XPS data of Ru/np-MoS_2. The XPS data of np-MoS_2 are also shown as a reference. (**d**) The compositions of np-MoS_2 and Ru/np-MoS_2 result from XPS analyses.

As shown in **Supplementary Figure 16b**, the Mo 3d peaks of np-MoS_2 shows the negative energy shifts as comparison with that of 2H-MoS_2 (**Ref. 5**), indicating the co-existence of 2H-MoS_2 and 1T-MoS_2 in np-MoS_2. After the introduction of Ru atoms, the Mo 3*d* peaks of Ru/np-MoS_2 displays the further negative energy shifts as comparison with that of np-MoS_2, suggesting the increase of 1T-MoS_2 in Ru/np-MoS_2.

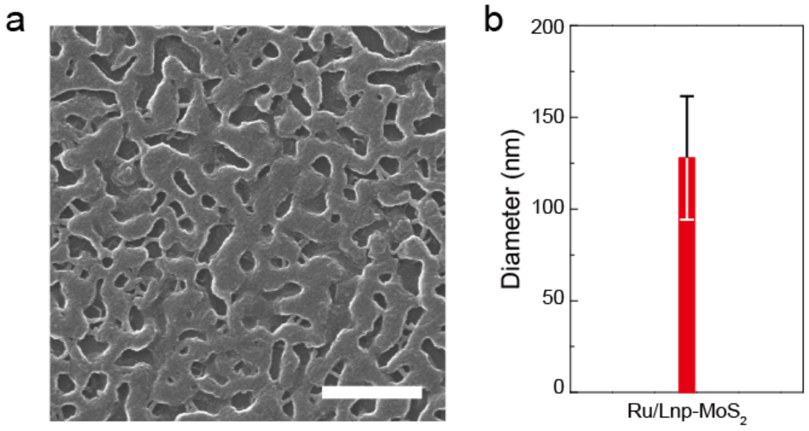

Supplementary Figure 17. SEM characterizations of Ru/Lnp-MoS_2.

(**a**) SEM image of Ru/Lnp-MoS_2. (**b**) The average diameters of ligament for Ru/Lnp-MoS_2. Error bars represent the standard deviation from multiple measurements. Scale bar: 500 nm.

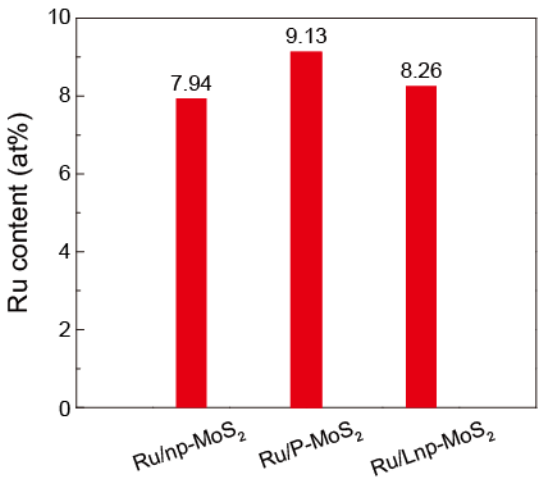

Supplementary Figure 18. Characterizations of Ru content.

The Ru contents of Ru/np-MoS_2, Ru/P-MoS_2, and Ru/Lnp-MoS_2 result from EDS analyses.

The Ru content in this work is relatively high compared with the works reported so far (**Supplementary Table 2**). For the common synthesis strategy of single-atom catalysts, the numbers of defect or anchoring ligand always limit the metal load. However, the isolated metal atoms are substitutional doped into the MoS_2 in the spontaneous reduction strategy, thus avoiding the limitation of the number of defect or anchoring ligand. This is main responsible for the high metal content in the catalysts prepared by spontaneous reduction strategy.

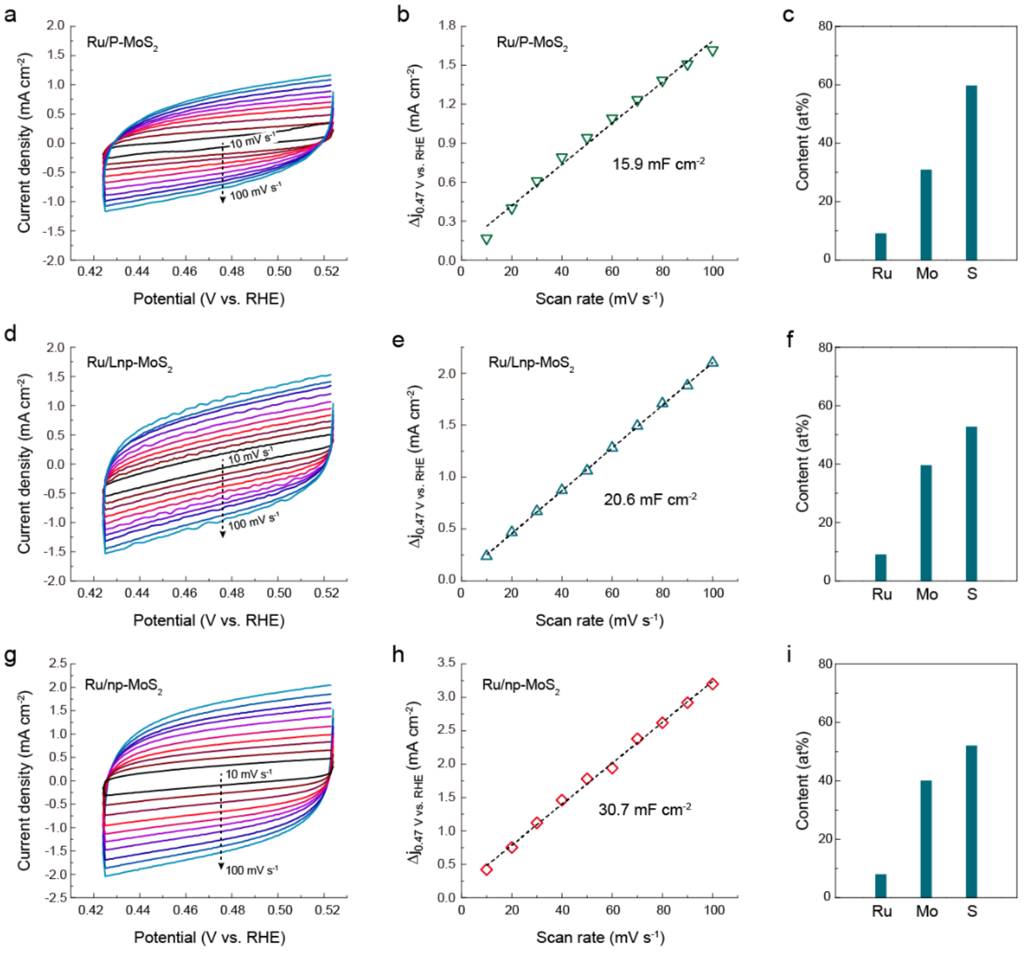

Supplementary Figure 19. Characterizations for Ru/P-MoS_2 and Ru/Lnp-MoS_2.

CVs of Ru/P-MoS_2 (**a**), Ru/Lnp-MoS_2 (**d**), and Ru/np-MoS_2 (**g**) measured in non-Faradaic region. (**b**, **e**, **h**) The corresponding plots of current densities against scan rates. Δj is the difference between anodic and cathodic current densities at a same potential. The half of the value of the slope is used as the double-layer capacitance of electrodes. (**c**, **f**, **i**) The compositions of Ru/P-MoS_2, Ru/Lnp-MoS_2, and Ru/np-MoS_2 resulting from EDS analyses.

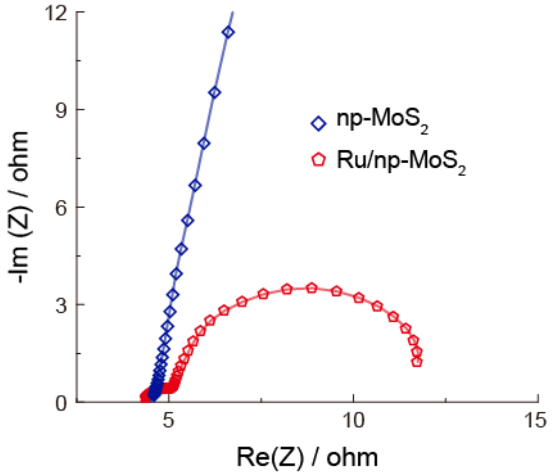

Supplementary Figure 20. Electrochemical impedance spectroscopy analyses.

Nyquist plots of np-MoS_2 and Ru/np-MoS_2 at the overpotential of 30 mV.

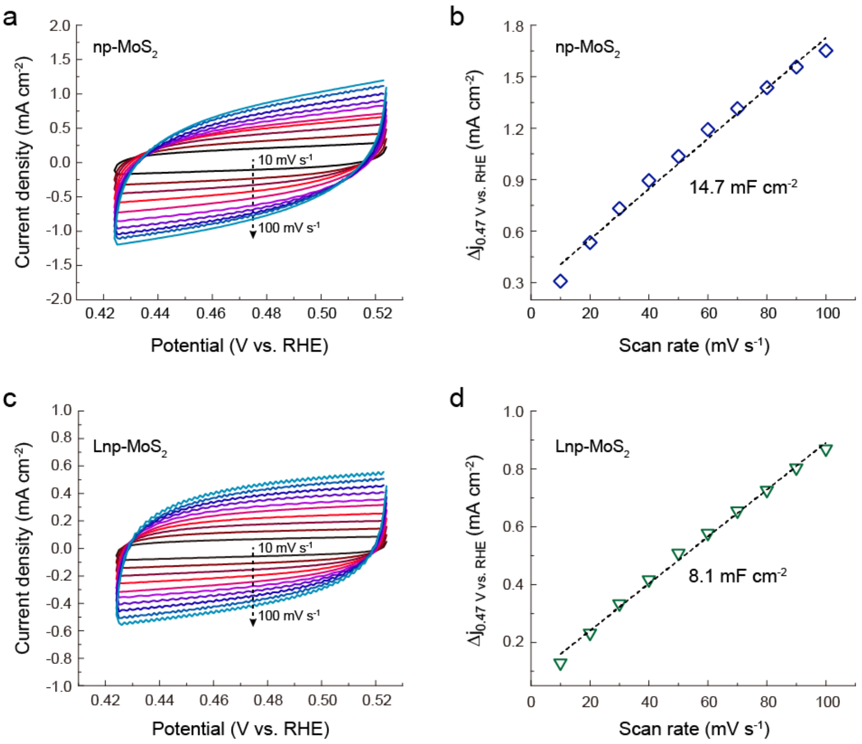

Supplementary Figure 21. Double-layer capacitance measurements.

CVs of np-MoS_2 (**a**) and Lnp-MoS_2 (**c**) measured in non-Faradaic region. (**b**, **d**) The corresponding plots of current densities against scan rates. Δj is the difference between anodic and cathodic current densities at a same potential. The half of the value of the slope is used as the double-layer capacitance of electrodes.

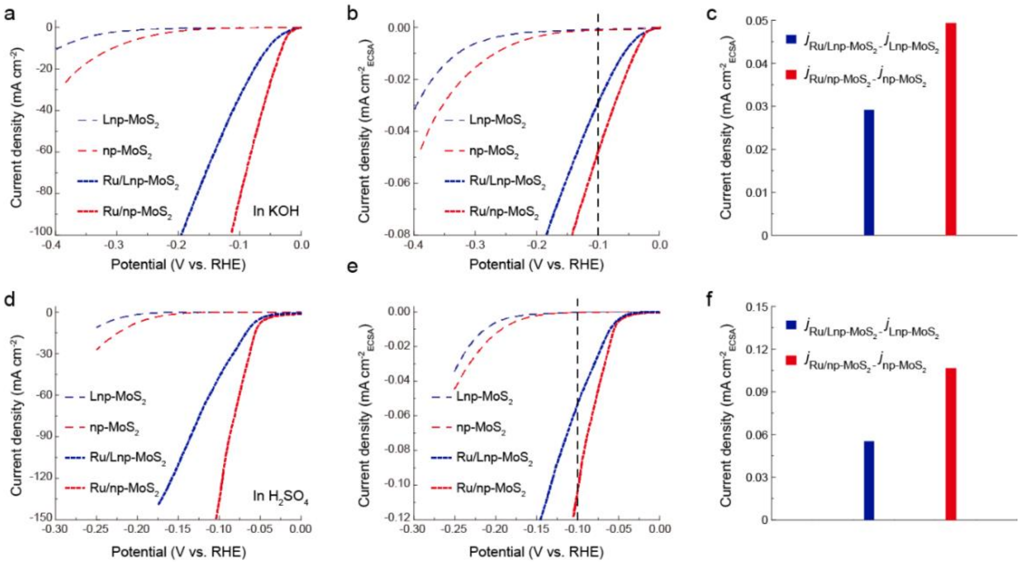

Supplementary Figure 22. Catalytic HER performances.

Polarization curves of Ru/Lnp-MoS_2 and Ru/np-MoS_2 as compared with that of Lnp-MoS_2 and np-MoS_2 in alkaline (**a**) and acidic (**d**) solutions. (**b**, **e**) Corresponding ECSA-normalized polarization curves. The increment of ECSA-normalized current density (at -0.10 V vs. RHE) after the formation of local Ru/1T-MoS_2 active structure for Ru/Lnp-MoS_2 and Ru/np-MoS_2 in alkaline (**c**) and acidic (**f**) solutions. *j* represents the current density.

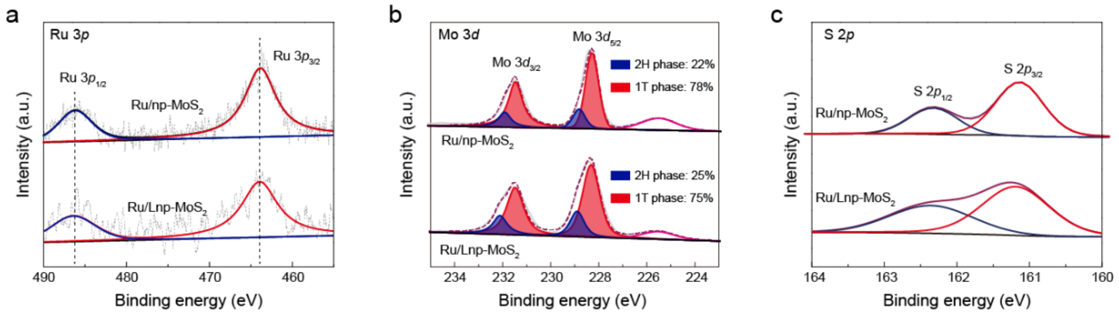

Supplementary Figure 23. XPS characterizations.

High-resolution Ru 3*p* (**a**), Mo 3*d* (**b**), and S 2*p* (**c**) XPS data of Ru/np-MoS_2 and Ru/Lnp-MoS_2.

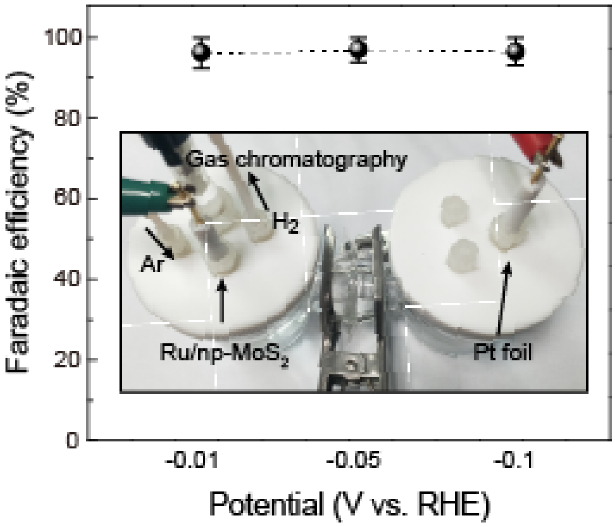

Supplementary Figure 24. Faradaic efficiency measurements for H_2 production.

Faradaic efficiencies of Ru/np-MoS_2 for H_2 production at different applied potentials. Inset shows the device for Faradaic efficiency measurements. Error bars represent the standard deviation from multiple measurements.

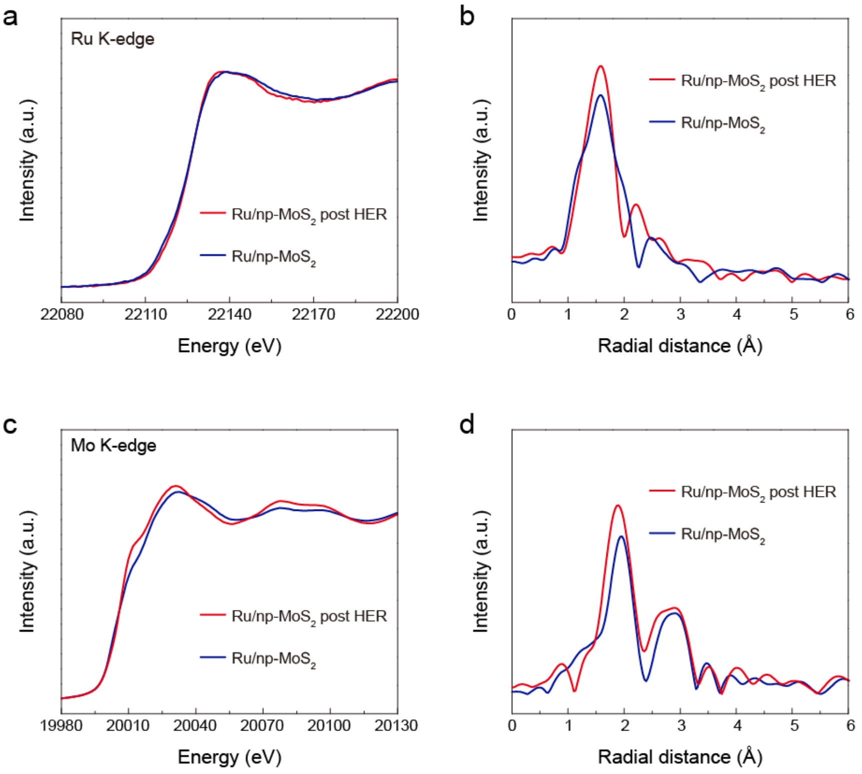

Supplementary Figure 25. XAS characterizations after long-time operation.

(**a, c**) XANES spectra at Ru K- and Mo K-edges. (**b, d**) Corresponding FT-EXAFS spectra at Ru K- and Mo K-edges.

The FT-EXAFS spectra at Ru K-edges show the negative-shift of Ru-Mo peak after long-time operation, indicating the shrinkage of interatomic distance between Ru atoms and Mo atoms. This irreversible shrinkage structure may be able to stabilize the isolated Ru atoms. The operation for a long-time also lead to the slight changes in oxidation state and structure of np-MoS_2, manifesting as the positive-shift of rising edge in XANES spectra at Mo-K edge and the low-R shift of Mo-S peak in corresponding FT-EXAFS spectra.

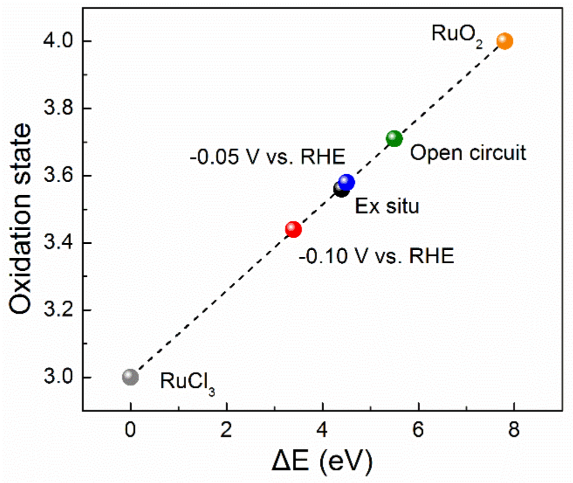

Supplementary Figure 26. The fitted average oxidation states.

The fitted average oxidation states of Ru from XANES spectra.

In the determining of oxidation state of Ru, we analyzed the absorption energy, which was obtained from the first maximum in the first-order derivative as the electron vacancy. The $RuCl_3$ (+3) and RuO_2 (+4) were used as the comparison standards.

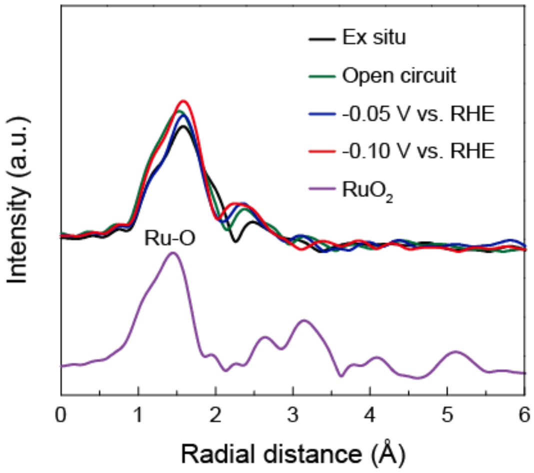

Supplementary Figure 27. Operando Ru K-edge FT-EXAFS spectra.

The FT-EXAFS spectra of Ru/np-MoS_2 recorded at different applied voltages.

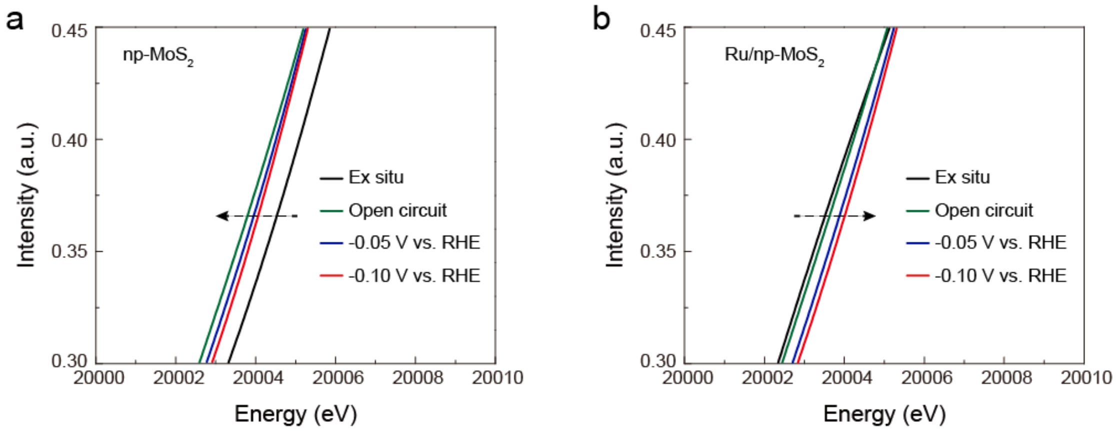

Supplementary Figure 28. Magnified rising edge XANES regions.

Magnified rising edge XANES regions recorded at the Mo K-edge of np-MoS_2 and Ru/np-MoS_2.

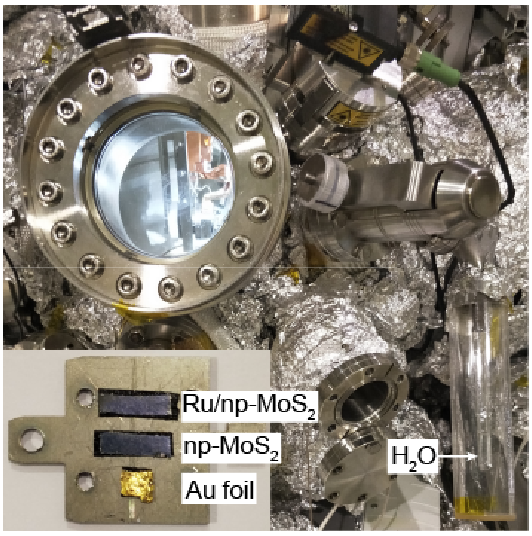

Supplementary Figure 29. The device for AP-XPS measurements.

AP-XPS measurements were performed at on the 24A1 beamline of NSRRC. In order to ensure the accuracy of the experiment, we prepared an np-MoS_2 material and cut it into two identical np-MoS_2 films (inset of **Supplementary Fig. 29**). One of them was used as an undoped sample (np-MoS_2) while another one was used as support for Ru doping (Ru/np-MoS_2). The Ru/np-MoS_2 film, np-MoS_2 film, and Au foil were directly covered on the carbon conductive adhesive, thus avoiding the influence of carbon conductive adhesive signal. Then, the sample holder loaded with Ru/np-MoS_2 film, np-MoS_2 film, and Au foil was exposed in the analysis chamber. The AP-XPS analyses were conducted under UHV, a water pressure of 0.01 torr, and a water pressure of 0.1 torr, respectively. The obtained XPS data were corrected by using the Au 4*f* XPS spectrum of Au foil.

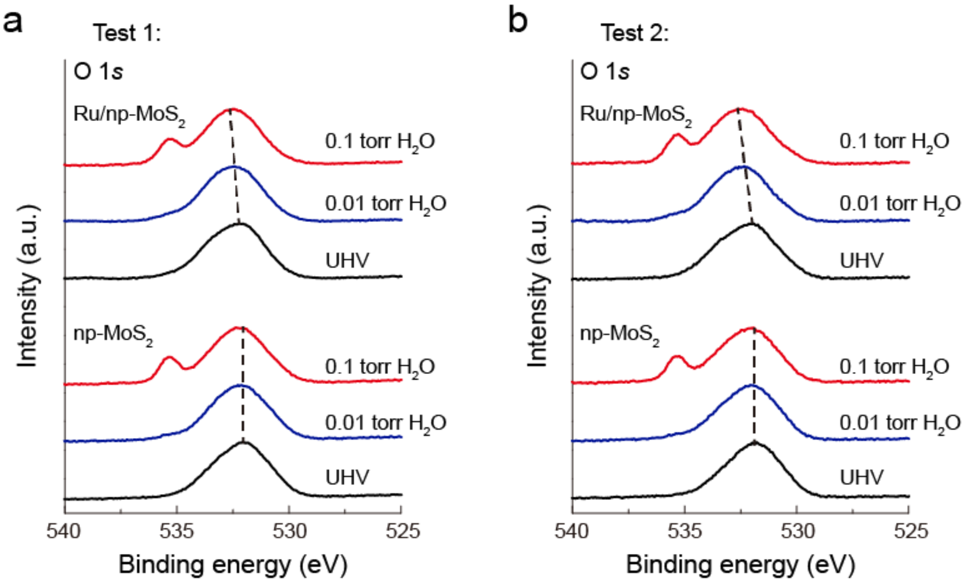

Supplementary Figure 30. AP-XPS measurements.

In order to acquire accurate results, we performed two AP-XPS measurements. Similar results were obtained in the two tests, which further proved the accuracy of the results. It is distinct that the O 1*s* XPS spectra of Ru/np-MoS_2 display obvious high-energy shifts with the increase of water pressure, resulting from the increase contribution of adsorbed water (Ads. H_2O) (**Fig. 5g**). Therefore, it is deduced that Ru/np-MoS_2 own stronger water affinity than np-MoS_2 due to the formation of strained SVs, which expose strained Mo sites as optimal water adsorption sites in Ru/np-MoS_2. Even though there are hydrophilic Ru atoms in Ru/np-MoS_2, Mo atoms contribute more water adsorption sites due to the low content of Ru atoms (Note that the H_2O adsorption energy of strained Mo sites is close to that of strained Ru sites, as shown in **Supplementary Fig. 2**).

Supplementary Note 1: DFT calculations.

DFT calculations were performed by using the Vienna Ab Initio Simulation Package (VASP). The projector augmented wave (PAW) method was conducted to describe/treat interactions between the valence electrons and the ion cores. The exchange correlation energy was modelled using Perdew-Burke-Ernzerhof (PBE) functional within the generalized gradient approximation (GGA). The cut-off energy was set to 450 eV and employed for the expansion of the wave functions. During structural optimization and the self-consistent calculations, the Brillouin zone (BZ) was sampled by a Monkhorst-Pack k-mesh of $5\times5\times1$. A vacuum layer of 20 Å was added perpendicular to the slab surface. The van der Waals interactions are taking account by employing DFT-D3 (BJ) method developed by S. Grimme et al. (**Ref. 6**, **7**). A unit cell ($4\times4\times1$) of 1T-MoS_2 was select to establish the model. For the Ru/1T-MoS_2 model, the Ru atom was exposed on the surface of 1T-MoS_2 by replacing a Mo atom and losing two S atoms. Usually, the adatom or dopant cannot change the lattice structure of the MoS_2, but causing local distortions. Thus, while we optimize the structures, we keep the lattice structure stable and optimize the positions of atoms. The unit cell was optimized until the force and total energy were set to be 0.01 eV/Å and 10^{-5} eV, respectively. For the application of strain, our preliminary work before this work suggests that the np-MoS_2 experience tensile strain (about 10%) originated from the nanotube-shaped ligament (**Ref. 8**, **9**). Ideally, the uniaxial tensile strain (10%) was applied on the above model. The subsequent HAADF-STEM

characterizations show that the value of tensile strain is about 12% (**Fig. 2g**).

The generally accepted alkaline HER mechanism consists of two steps, with the Volmer step followed by the Tafel step or Heyrovsky step:

$$\text{Volmer step: cat} + H_2O + e^- \rightarrow H^*@\text{cat} + OH^- \text{(Equation 1)}$$

$$\text{Tafel step: } 2H^*@\text{cat} \rightarrow H_2 \text{ (Equation 2)}$$

$$\text{Heyrovsky step: } H^*@\text{cat} + H_2O + e^- \rightarrow \text{cat} + OH^- + H_2 \text{ (Equation 3)}$$

The free energies of step (1) and step (3) should be the same at equilibrium potential. Computations on the exact free energy of OH^- in solutions could be avoided by using computational hydrogen electrode based on the above assumption (**Ref. 10**).

The free energies (ΔG) of steps 1-3 are calculated using the following equation adopted from **Ref. 11**:

$$\Delta G = \Delta E + \Delta ZPE + \Delta U(0 \rightarrow T) - T\Delta S \text{ (Equation 4)}$$

Where, ΔE, ΔZPE, $\Delta U(0 \rightarrow T)$, and $T\Delta S$ are the adsorption energy of adsorbed species on the given unit cell, the differences corresponding to the zero-point energy, the difference of the correction to the thermal energy, and the differences corresponding to the entropy (at 298.15 K), respectively. ΔZPE could be derived after frequency calculation by the following equation: (**Ref. 12**):

$$ZPE = \frac{1}{2}\sum hv_i \text{ (Equation 5)}$$

Where h is the Planck constant, v_i are the computed vibrational frequencies.

The thermal energy correction is calculated by the following equation:

$$U\,(0 \rightarrow T) = \sum \frac{hv_i}{\exp(\beta hv_i) - 1} \text{ (Equation 6)}$$

The TS values of adsorbed species are calculated in the following equation (**Ref. 11,**

13, 14):

$$TS^{vib}(T, v) = \sum \kappa T(\frac{\beta h v_i}{\exp(\beta h v_i) - 1} - \ln(1 - \exp(-\beta h v_i))) \text{ (Equation 7)}$$

Where $\beta = 1/\kappa T$, $\{v_i\}$ are vibrational modes, κ is the Boltzmann constant, T is the temperature (which is set to 298 K in the present work), respectively. In the present study, the Gibbs free energy is calculated using VASPKIT, and the frequencies below 50 cm^{-1} are approximately equal to 50 cm^{-1} during the calculations of the vibration contributions to the adsorbed molecular free energy correction (**Ref. 15**).

Supplementary Note 2: The syntheses of Ru/P-MoS_2 and Ru/Lnp-MoS_2

The P-MoS_2 was synthesized by using Au foil as substrates for the chemical vapor deposition of monolayer MoS_2. Then, the monolayer MoS_2@Au composites were etched by I_2-KI solution for 24 h to obtain the P-MoS_2. The Lnp-MoS_2 was synthesized by using the same method as np-MoS_2, excepting for increasing the heat preservation of NPG before the sulfidation. This leads to the coarsening of NPG ligament, thus providing the templates with large ligament for the growth of the monolayer MoS_2. Finally, Ru/P-MoS_2 and Ru/Lnp-MoS_2 with the same Ru load to Ru/np-MoS_2 were prepared by using the spontaneous reduction technique.

Supplementary Note 3: Calculation of ECSA.

The real surface area of catalysts for HER was calculated from the ECSA, which can be converted from the specific capacitance. The specific capacitance for a flat surface is generally found to be in the range of 20-60 μF cm^2_{geo}.

$$A_{ECSA} = \frac{\text{specific capacitance}}{60\ \mu F\ cm^2_{geo}\ \text{per}\ cm^2_{ECSA}}$$

The ECSA of Ru/np-MoS_2, Ru/P-MoS_2, and Ru/Lnp-MoS_2 were calculated as follow:

$$A_{ECSA}^{Ru/np-MoS_2} = \frac{15.35\ mF\ cm^{-2}}{60\ \mu F\ cm^2_{geo}\ \text{per}\ cm^2_{ECSA}} = 255.83\ cm^2_{ECSA}$$

$$A_{ECSA}^{Ru/P-MoS_2} = \frac{7.95\ mF\ cm^{-2}}{60\ \mu F\ cm^2_{geo}\ \text{per}\ cm^2_{ECSA}} = 132.50\ cm^2_{ECSA}$$

$$A_{ECSA}^{Ru/Lnp-MoS_2} = \frac{10.30\ mF\ cm^{-2}}{60\ \mu F\ cm^2_{geo}\ \text{per}\ cm^2_{ECSA}} = 171.67\ cm^2_{ECSA}$$

The ECSA of np-MoS_2 and Lnp-MoS_2 were calculated as follow:

$$A_{ECSA}^{np-MoS_2} = \frac{7.35\ mF\ cm^{-2}}{60\ \mu F\ cm^2_{geo}\ \text{per}\ cm^2_{ECSA}} = 122.50\ cm^2_{ECSA}$$

$$A_{ECSA}^{Lnp-MoS_2} = \frac{4.05\ mF\ cm^{-2}}{60\ \mu F\ cm^2_{geo}\ \text{per}\ cm^2_{ECSA}} = 67.5\ cm^2_{ECSA}$$

Supplementary Table 1. Key values for water dissociation and hydrogen generation on the surface of different models.

**Models**	**Δ*E*(H_2O)/eV**	**Δ*ZPE*(H_2O)+$U_{(0->T)}$(H_2O)/eV**	**Δ*TS*(H_2O)/eV**	**Δ*G*(H_2O)/eV**
Ru/MoS_2	0.295	0.590+0.102	0.236	0.751
Strained Ru/MoS_2	-0.154	0.602+0.064	0.128	0.384
**Models**	**Δ*E*(H*)/eV**	**Δ*ZPE*(H*)+$U_{(0->T)}$(H*)/eV**	**Δ*TS*(H*)/eV**	**Δ*G*(H*)/eV**
Ru sites	0.557	0.224+0.010	0.013	0.778
Mo sites	0.596	0.267+0.005	0.006	0.862
S sites	0.945	0.208+0.013	0.018	1.148
Strained Ru sites	-0.035	0.227+0.008	0.011	0.189
Strained Mo sites	0.651	0.175+0.008	0.012	0.822
Strained S sites	0.459	0.211+0.012	0.017	0.665

Supplementary Table 2. Structural parameters extracted from the Mo K-edge EXAFS fitting.

Catalysts	Scattering pair	*CN*	*R* (Å)	σ^2 (10^{-3} Å^2)	ΔE_0 (eV)	*R*-factor
P-MoS_2	Mo-S	5.1±0.7	2.400±0.01	1.55±0.8	0.272	0.005
	Mo-Mo	3.9±0.5	3.149±0.01	2.48±0.8	-2.14	
Lnp-MoS_2	Mo-S	5.9±0.5	2.405±0.01	2.63±0.6	2.53	0.008
	Mo-Mo	4.1±0.4	3.161±0.01	3.70±0.6	2.43	
np-MoS_2	Mo-S	5.7±0.7	2.408±0.01	2.66±1.3	2.77	0.010
	Mo-Mo	4.1±0.5	3.169±0.01	3.42±1.3	2.90	

CN represents the coordination number; *R* represents the interatomic distance; σ^2 represents the Debye-Waller factor; ΔE_0 represents the edge-energy shift.

Supplementary Table 3. Comparison of overpotential at 10 mA cm^{-2} and Tafel slope of Ru/np-MoS_2 with recently reported SACs and MoS_2-based catalysts.

**Catalysts**	**Metal content**	**Electrolyte**	**η@10 mA cm^{-2} (mV)**	**Tafel slope (mV dec^{-1})**	**Ref.**
Ru/np-MoS_2	Ru:7.94 at%	1.0 M KOH	30	31	This work
Ru/C	20 wt%	1.0 M KOH	100	69	This work
Pt/C	20 wt%	1.0 M KOH	40	46	This work
NiO@1T-MoS_2	Ni: 4.99 wt%	1.0 M KOH	46	52	16
C-MoS_2		1.0 M KOH	45	46	17
N, Mn-MoS_2	Mn: 3.49 at%	1.0 M KOH	66	50	18
Ir-MoS_2	Ir: 11.75 at%	1.0 M KOH	44	32	19
Ni-MoS_2	Ni: 13.3 at%	1.0 M KOH	98	60	20
Ru-MoS_2/CNT	Ru: ~5 at%	1.0 M KOH	50	62	21
$Ni(OH)_2/MoS_2$		1.0 M KOH	80	60	22
Pt@PCM	Pt: 0.53 wt%	1.0 M KOH	139	73.6	23
Ni NP|Ni-N-C/EG	Ni: 11.8 wt%	1.0 M KOH	147	114	24
SA-Ru-MoS_2	Ru: 2.29 at%	1.0 M KOH	76	21	25
Mo SAs/ML-MoS_2	Mo: 1.23 at%	1.0 M KOH	209	35.1	26
Co-MoS_2/BCCF-21	Co: 1.62 at%	1.0 M KOH	48	52	27
Pd, Ru-$MoS_{2-x}OH_y$	Ru: ~2 at%	1.0 M KOH	48	45	28

Supplementary References

1. Emery, A. A. & Wolverton, C. High-throughput DFT calculations of formation energy, stability and oxygen vacancy formation energy of ABO_3 perovskites. *Sci. Data* **4**, 170153 (2017).

2. Erlebacher, J. Evolution of nanoporosity in dealloying. *Nature* **410**, 450 (2001).

3. Song, I. et al. Patternable large-scale molybdenium disulfide atomic layers grown by gold-assisted chemical vapor deposition. *Angew. Chem. Int. Ed.* **53**, 1266-1269 (2014).

4. Li, H. et al. Systematic design of superaerophobic nanotube-array electrode comprised of transition-metal sulfides for overall water splitting. *Nat. Commun.* **9**, 2452 (2018).

5. Yu, Y. et al. High phase-purity 1T'-MoS_2- and 1T'-$MoSe_2$-layered crystals. *Nat. Chem.* **10**, 638-643 (2018).

6. Grimme, S. et al. A consistent and accurate ab initio parametrization of density functional dispersion correction (DFT-D) for the 94 elements H-Pu. *J. Chem. Phys.* **132**, 154104 (2010).

7. Grimme, S. et al. Effect of the damping function in dispersion corrected density functional theory. *J. Comp. Chem.* **32**, 1456 (2011).

8. Tan, Y. et al. Monolayer MoS_2 films supported by 3D nanoporous metals for high-efficiency electrocatalytic hydrogen production. *Adv. Mater.* **26**, 8023-8028 (2014).

9. Chen, D. et al. General synthesis of nanoporous 2D metal compounds with 3D bicontinous structure. *Adv. Mater.* **32**, 2004055 (2020).

10. Blöchl, P. E. Projector augmented-wave method. *Phys. Rev.* B **50**, 17953 (1994).

11. Reuter, K. & Scheffler, M. Composition, structure, and stability of RuO_2 (110) as a function of oxygen pressure. *Phys. Rev. B* **65**, 035406 (2001).

12. Zheng, Y. et al. High electrocatalytic hydrogen evolution activity of an anomalous ruthenium catalyst. *J. Am. Chem. Soc.* **138**, 16174-16181 (2016).

13. Nørskov, J. K. et al. Trends in the exchange current for hydrogen evolution. *J. Electrochem. Soc.* **152**, J23-J26 (2005).

14. Liu, Z. et al. Hole polaron diffusion in the final discharge product of lithium-sulfur batteries. *J. Phys. Chem. C* **121**, 17169-17175 (2017).

15 Wang, V. et al. VASPKIT: A user-friendly interface facilitating high-throughput computing and analysis using VASP code. arXiv: 1908. 08269 (2019).

16. Huang, Y. et al. Atomically engineering activation sites onto metallic 1T-MoS_2 catalysts for enhanced electrochemical hydrogen evolution. *Nat. Commun.* **10**, 982 (2019).

17. Zang, Y. et al. Tuning orbital orientation endows molybdenum disulfide with exceptional alkaline hydrogen evolution capability. *Nat. Commun.* **10**, 1217 (2019).

18. Sun, T. et al. Engineering the electronic structure of MoS_2 nanorods by N and Mn dopants for ultra-efficient hydrogen production. *ACS Catal.* **8**, 7585-7592 (2018).

19. Wei, S. et al. Iridium-triggered phase transition of MoS_2 nanosheets boosts overall water splitting in alkaline media. *ACS Energy Lett.* **4**, 368-374 (2018).

20. Zhang, J. et al. Engineering water dissociation sites in MoS_2 nanosheets for accelerated electrocatalytic hydrogen production. *Energy Environ. Sci.* **9**, 2789-2793 (2016).

21. Zhang, X. et al. Engineering MoS_2 basal planes for hydrogen evolution via synergistic ruthenium doping and nanocarbon hybridization. *Adv. Sci.* **6**, 1900090 (2019).

22. Zhang, B. et al. Interface engineering: The $Ni(OH)_2/MoS_2$ heterostructure for highly efficient alkaline hydrogen evolution. *Nano Energy* **37**, 74-80 (2017).

23. Zhang, H et al. Dynamic traction of lattice-confined platinum atoms into

mesoporous carbon matrix for hydrogen evolution reaction. *Sci. Adv.* **4**, eaao6657 (2018).

24. Lei, C. et al. Efficient alkaline hydrogen evolution on atomically dispersed Ni-Nx Species anchored porous carbon with embedded Ni nanoparticles by accelerating water dissociation kinetics. *Energy Environ. Sci.* **12**, 149-156 (2019).

25. Zhang, J. et al. Single-atom Ru doping induced phase transition of MoS_2 and S vacancy for hydrogen evolution reaction. *Small Methods* **3**, 1900653 (2019).

26. Luo, Y. et al. Unsaturated single atoms on monolayer transition metal dichalcogenides for ultrafast hydrogen evolution. *ACS Nano* **14**, 767-776 (2019).

27. Xiong, Q. et al. Cobalt covalent doping in MoS_2 to induce bifunctionality of overall water splitting. *Adv. Mater.* **30**, 1801450 (2018).

28. Luo, Z. et al. Reactant friendly hydrogen evolution interface based on di-anionic MoS_2 surface. *Nat. Commun.* **11**, 1116 (2020).
